# Tamoxifen represses alcohol-induced transcription of RNA polymerase III-dependent genes in breast cancer cells

**DOI:** 10.18632/oncotarget.2678

**Published:** 2014-11-04

**Authors:** Qian Zhong, Ganggang Shi, Qingsong Zhang, Lei Lu, Daniel Levy, Shuping Zhong

**Affiliations:** ^1^ Department of Biochemistry and Molecular Biology, Keck School of Medicine, University of Southern California, Los Angeles, CA, USA; ^2^ State Key Laboratory of Oncology in South China, Sun Yat-sen University Cancer Center, China; ^3^ Shantou University Medical College, Shantou, Guangdong, China

**Keywords:** Tamoxifen, Alcohol, Estrogen receptor, C-Jun, Brf1, Pol III genes, Breast cancer

## Abstract

Alcohol consumption in women has been associated with an increased risk of breast cancer, particular in estrogen receptor positive (ER+) cases. Deregulation of RNA polymerase III-dependent (Pol III) transcription enhances cellular tRNAs and 5S rRNA production, leading to an increase in translational capacity to promote cell transformation and tumor formation. Our recent studies demonstrated that alcohol induces Brf1 expression and Pol III gene transcription via ER. Here, we report that Tamoxifen (Tam) inhibits the induction of Brf1 and Pol III genes in ER+ breast cancer cells. Further analysis indicates that alcohol increases c-Jun expression to upregulate the transcription of Brf1 and Pol III genes, whereas Tam reduces c-Jun expression to repress the transcription of Brf1. Repression of cJun decreases cellular levels of ERα and Brf1. Alcohol-dependent increased occupancy of Brf1 in Pol III gene promoters is reduced by Tam. The repression of Brf1 and Pol III genes by Tam reduces alcohol-induced cell proliferation and colony formation. Together, these results indicate that Tam inhibits alcohol-induced Brf1 expression through c-Jun and ERα to downregulate Pol III gene transcription. Our studies uncover a new mechanism of Tam-treated ER+ breast cancer, by which Tam inhibits tumor growth through repressing Pol III gene transcription.

## INTRODUCTION

Tamoxifen (Tam) is an antagonist of the estrogen receptor (ER) in breast tissue, which competitively binds to ER, producing a nuclear complex that decreases DNA synthesis and inhibits estrogen effects. Tam is currently used for the treatment of both early and advanced ER+ breast cancer in women [1]. Tam causes cells to remain in the G_0_ and G_1_ phases of the cell cycle to repress cell proliferation. Studies have indicated that Tam takes part in the regulation of gene transcription, such as c-Jun and c-Fos [2]. However, it is not clear whether Tam affects transcription of RNA polymerase III-dependent genes (Pol III genes), a variety of untranslated RNAs, including tRNAs, 5S rRNAs, which control the translational and growth capacity of cells [3-4]. Deregulation of Pol III genes is tightly linked to tumor development. Brf1 (TFIIB-related factor 1) specifically regulates Pol III gene transcription. Brf1 and products of Pol III genes are elevated in both transformed and tumor cells suggesting that they play a crucial role in tumorigenesis. Oncogenic proteins stimulate Pol III gene transcription [4-7]; whereas tumor suppressors repress this transcription [4-8]. Consistent with this idea, enhanced Pol III gene transcription is required for oncogenic transformation [7, 9].

Alcohol consumption is consistently associated with increased risk for breast cancer in women [10-12]. This association involves the ER, which is over-expressed in approximately 70-80% of breast cancer cases [13-14]. Alcohol is known to promote mammary tumorigenesis [15-20]. Our studies indicate that enhancement of Brf1 and Pol III gene expression is correlated with tumor formation in alcohol-fed mice [21]. We demonstrated that ethanol caused increase in expression of c-Jun, which upregulates transcription of Brf1 and Pol III genes in liver cells [21]. Alcohol enhances Brf1 and Pol III gene expression via the ER pathway in MCF-7 cells [22]. Alcohol elevates ERα activity and inhibition of ERα reduces Pol III gene transcription [22]. Given that Tam represses AP-1 *(c-jun* and *c-fos*) activity and AP-1 modulates ERα expression [2,23], it implies that Tam may affect transcription of Pol III genes. Here, our studies demonstrate, for the first time, that Tam inhibits Brf1 expression and Pol III gene transcription via the c-Jun and ERα pathway to repress cell proliferation and transformation. These studies enhance our understandings of the mechanism of Tam treatment of ER+ breast cancer cases and provide a potential approach to improve the efficacy of Tam by the co-utilization of potential inhibitors of this pathway to repress Pol III gene transcription.

## RESULTS

### Tam represses Pol III gene transcription via the alteration of Brf1

**Fig.1:**
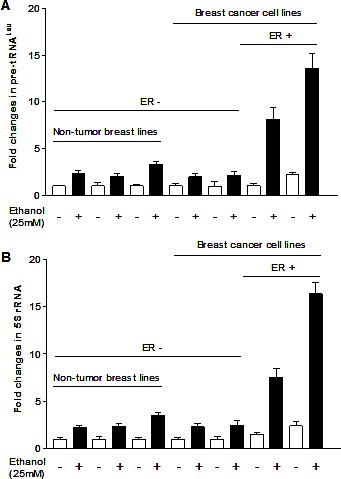
Alcohol induces RNA Pol III-dependent transcription ER+ breast cancer cell lines (MCF-7 and T47D), ER- breast cancer cell lines (MDA-MB231 and SKBR-3) and ER- non tumor cell lines (MCF-10F, MCF-12A and MCF-10A) were starved in FBS/DMEM-F12 for 3h. Cells were treated with or without 25 mM of ethanol. RNAs were isolated from these cells and RT-qPCR was performed to measure the amounts of pre-tRNA^Leu^ (A), 5S rRNA (B). The fold change was calculated by normalizing to the amount of GAPDH mRNA. The bars represent Mean ± SE of at least three independent determinations.

**Fig.2:**
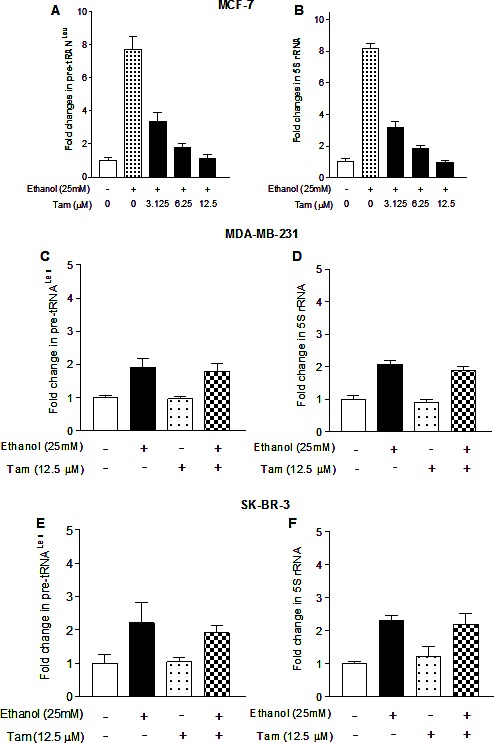
Tam represses RNA Pol III-dependent transcription (A and B): the cells of ER+ MCF-7 breast cancer lines were starved in DMEM-F12 for 3h. Cells were pretreated with different amounts of Tam for 1 hour, and then treated with 25 mM ethanol for another 1 h as indicated in Fig. 1. (C-F): the cells of ER- MDA-MB231 (C and D) and SKBR-3 (E and F) were pretreated with 12.5 μM Tam for 1 h and then treated with 25 mM ethanol. Total RNAs were extracted from these cells and RT-qPCR was performed to measure the amounts of pre-tRNA^Leu^ (A, C, E) and 5S rRNA (B, D, F). The fold change was calculated by normalizing to the amount of GAPDH mRNA. The bars represent Mean ± SE of at least three independent determinations.

Our study has demonstrated that alcohol induced RNA Pol III-dependent transcription *in vitro* and *in vivo* by using cell culture model and animal model [21]. Recent, we have reported that alcohol increases ERα expression to upregulate transcription of Pol III genes [22]. To investigate whether Tam affects Pol III gene transcription, human breast cells were treated with ethanol and the amounts of precursor tRNA^Leu^ and 5S rRNA transcript were measured by RT-qPCR. The results reveal that ethanol induces the transcription of Pol III genes, both pre-tRNA^Leu^ (Fig. 1A) and 5S rRNA (Fig. 1B), where the induction of Pol III genes in ER+ breast cancer cells lines (MCF-7 and T47D) is dramatically higher than in ER- breast cell lines, both cancer lines (MDA-MB231, SK-BR-3) and non-tumor lines (MCF-10A, MCF-10C and MCF-12A) (Fig. 1). These results demonstrate that alcohol-increased transcription of Pol III genes is associated with ERα expression. Tam is an antagonist of ER, which has widely been used in treatment of breast cancer. Given that alcohol increased ERα expression and reduction of ERα by its siRNA repressed Pol III gene activity [22], this implies that Tam may affect the Pol III genes. The results show that Tam treatment markedly inhibits the induction of pre-tRNA^Leu^ (Fig. 2A) or 5S rRNA (Fig. 2B) of MCF-7 cells by alcohol, but does not affect TFIIIC_63_, a non-Pol III-dependent gene (S1). This inhibition of Pol III genes by Tam is concentration-dependent and peaks at 12.5 μM Tam for 1 hour (h). Thus, this condition was used for the entire study unless stated otherwise. We then assessed the effect of Tam on Pol III genes in other breast cancer cell lines. The results indicate that Tam does not affect transcription of Pol III genes in ER- breast cancer cell lines of MDA-MB231 (Fig. 2C and 2D) and SK-BR-3 (Fig. 2E and 2F). Tam does not significantly affect Pol III gene transcription in MCF-7 cells without alcohol treatment (data not shown). These results support the idea that Tam represses Pol III gene transcription in an ER-dependent manner.

**Fig.3:**
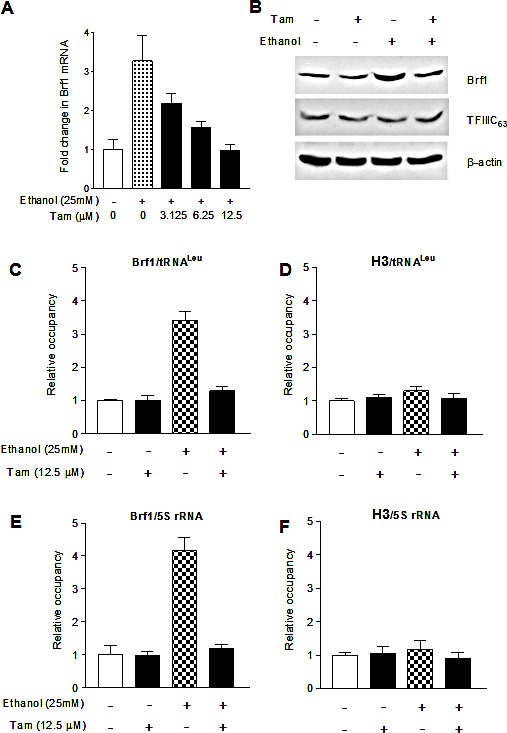
Tam reduces Brf1 expression and lowers the occupancy of Brf1 in the promoters of Pol III genes (A-B) *Tam decreased Brf1 expression.* MCF-7 cells were starved in DMEM-F12 for 3 h and treated with Tam as indicated in Fig. 2A. The total RNA and cell lysates from these cells were extracted to determine mRNA and protein of Brf1 (A and B) and TFIIIC63 (S1) by RT-qPCT and immunoblot analysis as described previously [22]. (C-F) *Tam-lowered Brf1 binding to the promoters.* MCF-7 cells were treated with Tam as described above. ChIP assays were performed using Brf1 (C and E) and histone H3 (D and F) antibodies and qPCR to quantify the amplified DNA as described previous [22]. The relative occupancy of the proteins was calculated based on the control (no Tam treatment). All values shown are the means ± SE of at least three independent chromatin preparations.

Brf1 is a key transcription factor regulating tRNA and 5S rRNA genes. Repressing Brf1 decreases Pol III gene transcription [22,24,25]. Therefore, we further determined whether Tam alters Brf1 expression. The results indicate that Tam treatment decreases cellular levels of Brf1 mRNA and protein (Fig. 3A and 3B). To explore how Tam affects Pol III gene transcription, we performed chromatin immunoprecipitation (ChIP) assay. The results indicate that Tam reduces the occupancy of Brf1 in the promoters of tRNA^Leu^ and 5S rRNA (Fig. 3C and 3E), compared to control of H3 (Fig. 3D and 3F). This indicates that Tam repress Pol III gene transcription through its inhibition of Brf1 expression.

### Reduction of c-Jun expression affects alcohol-induced Pol III gene transcription

**Fig.4:**
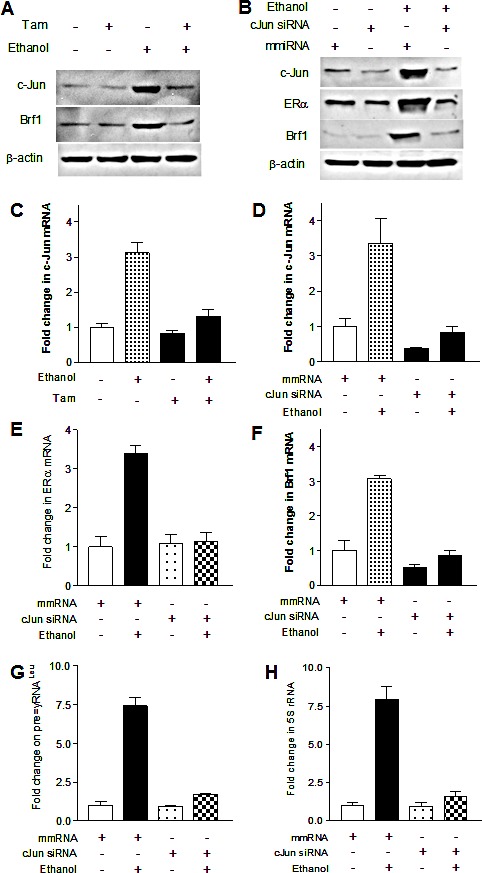
Down-regulating c-Jun decreases expression of Brf1 and Pol III genes (A and C) *Decrease in c-Jun expression* MCF-7 cells were treated as described in Fig.2. The cellular levels of c-Jun protein (A) and mRNA (C) were determined. Tam reduces the expression of c-Jun increased by alcohol. (B, D, E and F); *c-jun siRNA* MCF-7 cells were transfected with mismatch RNA (mmRNA) or c-Jun siRNA for 48 h. The cells were treated as described in A. RT-qPCR and immunoblot analysis was performed to determine cellular levels of c-Jun (B and D), ERα (B and E), and Brf1 (B and F); (G-H) *Pol III gene transcription* The cells were treated as described above. Total RNAs were extracted from these cells and RT-qPCR was performed to measure the amounts of pre-tRNA^Leu^ (G) and 5S rRNA (H). The fold change was calculated by normalizing to the amount of GAPDH mRNA. The bars represent Mean ± SE of at least three independent determinations.

**Fig.5:**
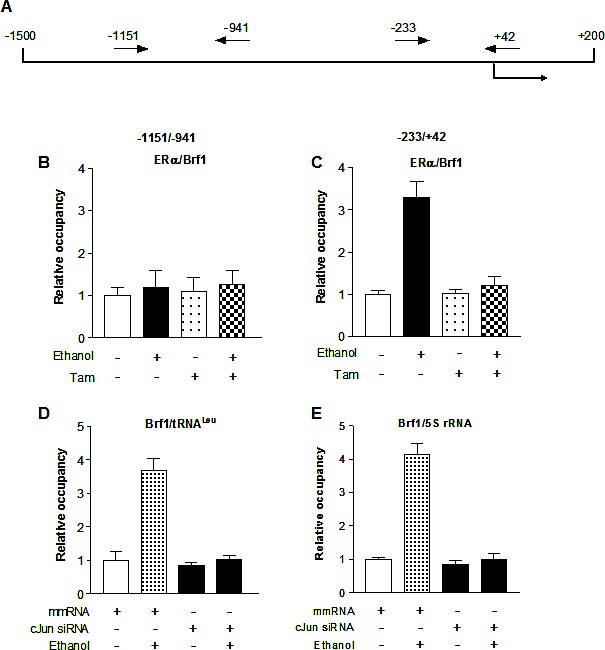
Tam reduces ERα binding to the promoters of Brf1 **(A-C)** *ERα binding to Brf1 promoter.* Schematic of the Brf1 promoter and primers used for ChIP assays are designated relative to the ERα site (A). MCF-7 cells treated with Tam and alcohol as described above to extract chromatin. ChIP assay were performed with ERα antibody to determine occupancy of ERα in the Brf1 promoter; (D-E) *Brf1 binding to the promoters of Pol III genes* The cells were treated as described above. ChIP assay were performed with Brf1 antibody and qPCR to quantify the amplified DNA of tRNA^Leu^ (D) and 5S rRNA (E). The relative occupancy of the proteins was calculated based on the control (no Tam treatment). All values shown are the means ± SE of at least three independent experiments.

As alcohol increases the c-Jun expression to elevate Brf1 and Pol III gene transcription in liver cells [21], we examine whether Tam affects the induction of c-Jun caused by alcohol in MCF-7 cells. The results reveal that alcohol increases c-Jun expression in MCF-7 cells, whereas Tam treatment reduces cellular levels of c-Jun protein and mRNA (Fig. 4A and 4C). Therefore, we further analyze how Tam changes Brf1 expression. The results indicate that repression of c-Jun by its siRNA decreases cellular levels of c-Jun protein (Fig. 4B) and mRNA (Fig. 4D). Further analysis indicates that repression of c-Jun by its siRNA reduces the levels of proteins and mRNAs of ERα (Fig. 4B and Fig. 4E) and Brf1 (Fig. 4B and 4F). Reduction of c-Jun by its siRNA also decreases alcohol-induced transcription of tRNA^Leu^ (Fig. 4G) and 5S rRNA (Fig. 4H). Next, we investigated how Tam affects Brf1 expression. We performed ChIP assays to determine if Tam affects occupancy of ERα in the Brf1 promoter (Fig.5A). The results reveal that ethanol increases the occupancy of ERα in Brf1 promoters near its transcription start site (TSS) in MCF-7 cells (Fig. 5C), but not in upstream of the TSS (Fig. 5B). It suggests that ERα is able to directly regulate Brf1 to modulate Pol III gene transcription [22], whereas Tam treatment inhibits the ability of ERα binding to Brf1 promoter (Fig. 5C). It suggests that Brf1 may be a target of Tam. Furthermore, the repression of c-Jun by its siRNA results in decrease in occupancy of Brf1 in the promoters of tRNA^Leu^ (Fig. 5D) and 5S rRNA (Fig. 5E), leading to downregulation of Pol III genes [22,24,25]. This implies that the alteration of c-Jun by Tam is important for alcohol-increased expression of Brf1 and Pol III genes in ER+ breast cancer cells.

### Tam decreases the rates of proliferation and transformation of MCF-7 cells

**Fig.6:**
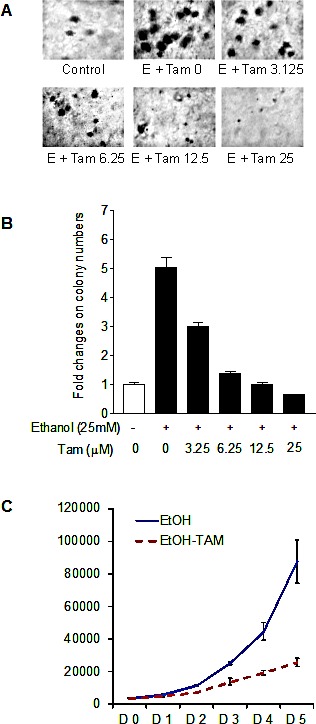
Tam caused phenotypic alteration induced by alcohol (A and B) *colony formation* MCF-7 cells were poured in triplicate into 6-well plate with 0.35% agar containing Tam and grown in the medium with different amounts of Tam or/and 25 mM ethanol. The cells were analyzed for colony formation in soft agar. (C). *cell proliferation* MCF-7 cells were poured into 6-well plate and cultured with 12.5 μM Tam or/and 25 mM ethanol. The viability and total cell numbers were measured daily for 5 days as described before [25].

Previous studies demonstrated that decreasing expression of Brf1 and Pol III genes was sufficient for repression of cell transformation [9,22,24]. Inhibition of Brf1 expression reduced anchorage-independent colonies to form and promoted tumor formation in mouse [9]. Our recent studies have further demonstrated that repression of Brf1 expression and Pol III gene transcription is able to inhibit alcohol-induced colony formation [22]. To further assess the effect of Tam on alcohol-caused phenotypic alteration, we perform a soft agar assay. The results show that alcohol enhances the rate of colony formation of MCF-7 cells (Fig. 6A), whereas Tam represses the cell anchorage-independent growth in a dose-dependent manner (Fig. 6A and 6B). The results of MCF-7 cell growth curve indicate that Tam lowers the rate of alcohol-induced cell growth (Fig. 6C). This indicates that Tam represses expression of Brf1 and Pol III genes, resulting in alteration of alcohol-promoted phenotypes of ER+ breast cancer cells.

## DISCUSSION

In this study, we perform a mechanistic analysis characterizing that Tam represses alcohol-caused induction of Brf1 and Pol III genes in ER+ breast cancer cells, resulting in decreasing the rate of cell proliferation and colony formation. Further analysis indicates that Tam decreases c-Jun expression and lowers alcohol-increased transcription of Pol III genes, which is correlated with ERα expression. The ability of Tam repressing Pol III gene transcription may have important implications for the development of Pol III gene lowering medications by using possible inhibitors of c-Jun or Brf1 to improve the efficacy of Tam treatment of breast cancer.

Epidemiologic studies have demonstrated that alcohol consumption has consistently been associated with an increased risk for breast cancer in both premenopausal and postmenopausal women [12-13]. Studies by Wang *et al* have demonstrated that alcohol increased MCP-1 and CRR2 expression, which promoted mammary tumor growth in alcohol-fed mice [18]. Alcohol intake is associated with ER+ breast cancer cases more than to ER- cases [14-15,26]. A recent study indicates that alcohol increased ERα expression to promote breast tumor formation in mice [19]. A previous study demonstrated that alcohol down-regulated the expression of BRCA1*,* a potent inhibitor of ERα, thereby contributing to breast cancer [26]. Alcohol intake was also shown to increase the transcriptional activity of ERα [27], as well as level of AP-1 expression [28]. We established that alcohol treatment increased c-Jun, a subunit of AP-1, expression and enhanced occupancy of TBP, Brf1 and tRNA^Leu^ promoters by c-Jun to elevate Pol III gene transcription in HepG2-ADH cells [21]. Studies from our and other laboratories have demonstrated that ethanol induces JNK1 activation in MCF-7 cells [22,29]. We have recently reported that alcohol activates JNK1 in MCF-7 cells to increase ERα expression, whereas inhibition of JNK1 represses ERα [22]. C-Jun is downstream target of JNK. This indicates that alcohol affects ERα expression through c-Jun pathway to affect Brf1 and Pol III gene transcription. The result is consistent with this study, where alcohol induced JNK1 activation to increase Pol III gene transcription in HepG2-ADH cells [21]. Our previous studies have demonstrated that JNK1 positively mediates Pol III gene transcription [30]. At the present study, the results indicate that alcohol increases cellular levels of c-Jun to upregulate Brf1 expression and Pol III gene transcription in MCF-7 cells. This implies that alcohol-enhanced c-Jun in both breast and liver cells may be a common signaling pathway to mediate Pol III gene transcription.

Our studies have demonstrated that EGF increased TFIIIB subunit, such as TBP, Brf1 and Bdp1, expression and enhanced Pol III gene transcription in JB6 cells [24]. Regulation of Bdp1, but not Brf1, occurred through JNK1-mediated alterations in TBP expression [30], suggesting that Brf1 and Bdp1 may be regulated independently. Alcohol induced Pol III gene transcription *in vivo* and *in vitro,* where this induction promoted tumor development in liver of NS5A transgenic mouse [21]. This indicates that deregulation of Pol III genes by alcohol promotes liver tumor development. Our recent studies have revealed that alcohol increases ERα activity which specifically modulates alcohol-induced Brf1 expression, but not TBP [22]. Studies have demonstrated that oncogenic proteins or tumor suppressors interacted with TFIIIB to enhance or repress Pol III gene transcription [4-8]. TBP interacts with the N-terminal activation domain of ERα, where it can induce and/or stabilized an ordered structure in the N-terminal regions of ERα [31]. This indicates that ERα does not affect TBP expression, while the interaction between ERα and TBP may mediate Pol III gene transcription. In contrast, change in cellular level of ERα by ethanol caused an alteration of Brf1 expression. ERα directly occupies the Brf1 promoter to modulate its expression. This finding is consistent with our recent findings that Brf1 is overexpressed in the human tissues of breast cancer (data not shown) and a previous study using human breast cancer biopsies, in which Brf1 expression in ER+ breast cancer cases is higher than in ER- cases [32]. This shows that ERα plays a critical important role in alcohol-induced deregulation of Pol III genes and alcohol-associated ER+ breast cancer. It suggests that Tam may affect the transcription of RNA Pol III genes induced by alcohol. However, nothing is known about the effects of Tam on Pol III gene transcription. Tam is currently used for the treatment of both early and advanced ER+ breast cancer in women [1]. Tam causes cells to remain in the G_0_ and G_1_ phases of the cell cycle to repress cell proliferation. Studies have indicated that Tam takes part in the regulation of gene transcription, such as c-*jun* and c-*fos* [2]. Given that Tam is an antagonist of the estrogen receptor in breast tissue and competitively binds to ER, we explore the role of Tam in alcohol-induced Pol III gene transcription. Our results indicate that Tam reduces cellular level of c-Jun, leading to decreases in ERα and Brf1 to repress Pol III gene transcription. These studies increase our understandings about the role of Tam: Tam is not only competitively binds to ER to inhibit ERα activity, but also reduce alcohol-increased c-Jun expression to decrease ERα expression, resulting in repression of Brf1 and Pol III genes. The present study also suggests that Brf1 may be a novel target of Tam. The Tam-mediated alteration of Brf1 expression may play an important role in alcohol-associated ER+ breast cancer.

In summary, the present study provides evidence that alcohol-induced increases in c-Jun activity enhances ERα expression, increasing ERα occupancy in the Brf1 promoter to enhance Brf1 expression, resulting in elevating Pol III gene transcription. Tam reduces cellular level of c-Jun to decrease the induction of Pol III gene transcription, resulting in inhibition of alcohol-induced cell proliferation and colony formation. Our studies uncover e new mechanism of Tam-treated ER+ breast cancer cases. It may explain the efficacy of Tam treatment in ER+ breast cancer cases. This is the first report that Tam suppresses RNA Pol III-dependent transcription induced by alcohol. The novel findings suggest the possibility that inhibition of Brf1 expression may be a potential approach to repress alcohol-promoted cell transformation and breast cancer development and to increase the efficacy of Tam treatment in Tam-resistant cases of ER+ breast cancer.

## MATERIALS AND METHODS

### Cell lines, reagents and antibodies

ER- human breast non-tumorigenic epithelial cell lines (MCF-10A, MCF-10F and MCF-10-2A), ER+ human breast cancer cell lines (MCF-7 and T-47D) and ER- human breast cancer cell lines MDA-MB231 and SKBR-3) were from ATCC (Manassas, Virginia, USA). Tamoxifen was from Sigma-Aldrich. Cell culture medium (DMEM/F12), OPTI-MEM, Lipofectamine 2000 and TRIzol reagent were from Life Technologies (San Diego, CA, USA). Antibodies against β-actin and TFIIIC_63_ and c-Jun siRNA (Catalog No. SC-29224) were obtained from Santa Cruz Biotech (Santa Cruz, CA, USA). Mismatch RNA was described previously [24]. Histone H3 antibody were from Cell Signaling (Danvers, MA, USA). Brf1 antibody was from Bethyl laboratories Inc (Montgomery, TX, USA). The sequences of primers were described in (Supplements) [8, 30].

### Real time quantitative PCR (RT-qPCR) and transfection

The cells of human breast cancer lines and non-tumor cell lines were grown to 85% confluence and starved in serum-free for 3 h. The cells were pretreated with Tam for 1 h and then treated with ethanol for another 1 h. Total RNA of these cells were extracted with TRIzol reagent (Invitrogen). For siRNA transfection assays, MCF-7 cells were cultured in 10% FBS/DMEM-F12 medium as described previously [22]. Serum-free medium was added to each dish with Lipofectamine2000-c-Jun siRNA or mismatch RNA complexes, and cells were further incubated for 4 h at 37◦C. The medium was changed with 10% FBS/DMEM-F12 and cells were incubated for 48 h before harvesting. Total RNA samples were quantified and reverse-transcribed in a 20 μl reaction containing 1 × RT (reverse transcription) buffer. After first-strand cDNA synthesis, the cDNAs were diluted in DNase-free water and real time qPCR (RT-qPCR) were performed with specific primers (Table S1) and PCR reagent kits (Bio-Rad Biotech) in the ABI prism 7700 Sequence Detection System. Precursor of tRNA^Leu^ and 5S rRNA transcripts and Brf1 and TFIIIC_63_ mRNA were measured by real time qPCR as described previously [22].

### Cell proliferation and anchorage-independent growth

Approximately 2 × 10^3^ MCF-7 cells were seeded in 6 well plates in triplicate. The cells were treated with 12.5 μM Tam and 25 mM ethanol. The cells were assayed for viability and counted each day for 5 days using a Coulter Counter [25].

MCF-7 cells (1 × 10^4^ cells/well in 6-well plate) were suspended in 0.35% (w/v) agar in 10% FBS/DMEM/F12 with or without 12.5 μM Tam, 25mM ethanol or both Tam and ethanol over a bottom layer of media with 0.5% (w/v) agar. Cells were fed fresh complete media with Tam or/and ethanol twice weekly. Colonies were counted 2-3 weeks or longer after plating as previously described [22].

### Immunoblot analysis

Cells were grown to 85% confluence in 10% FBS/DMEM and then serum deprived using DMEM for 4 h. Cells were treated with Tam for 1 h to extract total cell lysates. Protein concentrations of the resultant lysates were measured by the Bradford method using Fluostar Omega spectrometer (Cell Biology Core Laboratory of University of Southern California Research Center for Liver Diseases, P30 DK48522). Lysates (50 μg of protein) were subjected to immunoblot analysis as previously described [22]. Membranes were probed with specific antibodies as indicated. Hybond-P membrane was used for protein transfer. Bound primary antibody was visualized using horseradish peroxidase-conjugated secondary antibody (Vector Laboratories) and enhanced chemiluminescence reagents (Amersham).

### Chromatin immunoprecipitation (ChIP) assays

MCF-7 cells (3 × 10^6^ cells) were cultured in 15 cm dishes and treated with Tam or/and ethanol. The cells were fixed with formaldehyde (1% final concentration) at 24°C for 10 min. Soluble chromatin were prepared as described previously [25]. The chromatin were then diluted 1:10 with buffer (0.01% SDS, 1.1% Triton X-100, 1.2 mM EDTA, 16.7 mM Tris−HCl and 167 mM NaCl) and were subjected to immunoprecipitation (IP) in lysis buffer (50 mM Tris−HCl, 10 mM EDTA, 1% SDS) and a protease inhibitor cocktail set (CalBiochem). Pre-immune serum was used as a control and antibodies of Brf1, ERα or histone H3 were used for IP. The chromatin and antibodies were incubated at 4°C overnight. Complex of chromatin/antibody were recovered by adding 45 μl of protein A/G PLUS−agarose beads and incubated at 4°C for 2 h. The beads were sequentially washed for 10 min each in 1 ml of low salt, high salt and LiCl immune complex wash buffer. Immunocomplexes were eluted off the beads by incubation with 200 μl of 1% SDS and 50 mM NaHCO_3_. The eluents were incubated at 65°C for 6 h to reverse the formaldehyde-induced protein-DNA crosslinks. Extracted DNAs were resuspended in 100 μl of TE and qPCR were performed for amplification [4]. The primer sequences that were used are shown in Table S2 in Supplementary Data. The fold change in promoter occupancy was calculated by setting the level of promoter occupancy in the cells without ethanol treatment at 1.

## SUPPLEMENTARY MATERIAL FIGURE AND TABLES


