# Supplementary material for: Tamoxifen represses alcohol-induced transcription of RNA polymerase III-dependent genes in breast cancer cells
Source: Oncotarget. 2014 Nov 4;5(23):12410–7. doi: 10.18632/oncotarget.2678 (PMC4322994; doi:10.18632/oncotarget.2678)
Supplement: Supplementary file 1 [file oncotarget-05-12410-s001.pdf]

Tamoxifen represses alcohol-induced transcription of RNA polymerase III-dependent genes in breast cancer cells

Supplementary Material

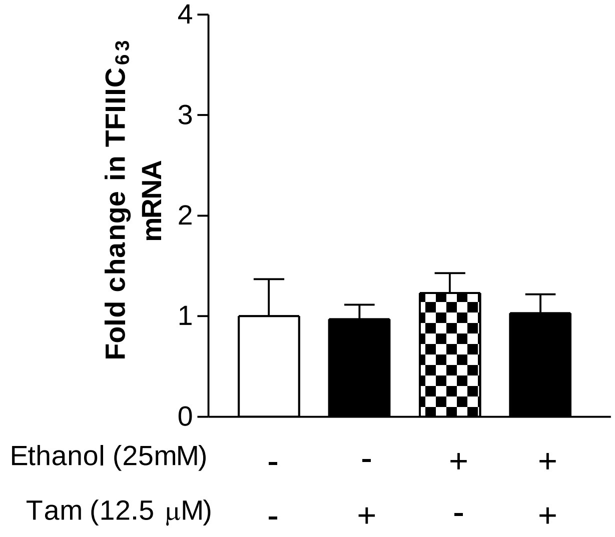

S. 1: Tam does not affect TFIIIC63 gene transcription. ER+ MCF-7 cells were starved in DMEM/12 for 3 h, pretreated with 12.5 μM Tam for 1 h, and then treated with 25 mM ethanol for another 1 h as indicated in **Fig. 1**. RNA was isolated from these cells and RT-qPCR was performed to measure the amounts $TFIIIC_{63}$ transcription. The fold change was calculated by normalizing to the amount of GAPDH mRNA. The bars represent Mean $\pm$ SE of at least three independent determinations.

Table S1: Primer Sets for Quantitative RT-PCR

**Target**	**Primers**	**Annealing Temperature**
Pre-tRNA^{Leu} (1)	(F) 5'-GTC AGG ATG GCC GAG TGG TCT AAG-3'<br>(R) 5'-CCA CGC CTC CAT ACG GAG AAC CAG AAG ACC C-3'	61º
5S rRNA (2)	(F) 5′ GGC CAT ACC ACC CTG AAC GC 3′<br>(R) 5′ CAG CAC CCG GTA TTC CCA GG 3′	61º
Human Brf1 (3)	(F) 5’ CCT CGG GCC TCT GCG GAG CAG -3’<br>(R) 5’ TCA TCA ATG GTC AAC TGA CTG GTG G -3’	60º

GAPDH (2)	(F) 5′-TCC ACC ACC CTG TTG CTG TA-3′<br>(R) 5′-ACC ACA GTC CAT GCC ATC AC-3′	61º

Abbreviations: (F) = forward, (R) = reverse.

Table S2: Primer Sets for ChIP

**Target**	**Primers**	**Annealing Temperature**
hBrf1 promoter –233/+42 (3)	(F) 5' CGT CCA GCT TTA GTC CCC GAC 3'<br>(R) 5' CGA GCC CAA GGC GGC TTC G 3'	63º
hBrf1 promoter –1153/–941	(F) 5' GAG ACA GAG TTT CAC TAT TGT C 3'<br>(R) 5' GGT ACA ACC GGT CCG ACC AGA GC 3'	62º
$tRNA^{Leu}$ gene (1)	(F) 5'-GTC AGG ATG GCC GAG TGG TCT AAG-3'<br>(R) 5'-CCA CGC CTC CAT ACG GAG AAC CAG AAG ACC C-3'	61º
5S rRNA (2)	(F) 5′ GGC CAT ACC ACC CTG AAC GC 3′<br>(R) 5′ CAG CAC CCG GTA TTC CCA GG 3′	61º

Supplemental references

1. Crighton D, Woiwode A, Zhang C, Mandavia N, Morton JP, Warnock LJ, *et al*., p53 represses RNA polymerase III transcription by targeting TBP and inhibiting promoter occupancy by TFIIIB. *EMBO J.* 2003; 22: 2810–2820.

2. Winter AG, Sourvinos G, Allison SJ, Tosh K, Scott PH, Spandidos DA, *et al*. RNA polymerase III transcription factor TFIIIC2 is overexpressed in ovarian tumors. *Proc Natl Acad Sci U S A.* 2000; 97: 12619-12624.

3. Zhang Q, Jin J, Zhong Q, Yu XL, Levy D, Zhong S. ERα mediates alcohol-induced deregulation of Pol III genes in breast cancer cells. *Carcinogenesis* 2013; 34: 28-37.
